# Design and Validation of a Laboratory-Scale System for Investigating Coking Byproducts

**DOI:** 10.1021/acsomega.5c06722

**Published:** 2025-10-16

**Authors:** Christian Manera, Guilherme Liziero Ruggio Da Silva, Bruno Deves Flores, Eduardo Osório, Marcelo Godinho, Antônio Cezar Faria Vilela

**Affiliations:** † Graduate Program in Mining, Metallurgical and Materials Engineering, 28124 Federal University of Rio Grande do Sul (UFRGS), Porto Alegre 90040-060, Rio Grande do Sul, Brazil; ‡ 339978 Gerdau Ouro Branco, Ouro Branco 36420000, Minas Gerais, Brazil; § Graduate Program in Process Engineering and Technologies, 58802 University of Caxias do Sul (UCS), Caxias do Sul 95070-560, Rio Grande do Sul, Brazil

## Abstract

The byproducts of metallurgical coke production play a significant role in the economic well-being of integrated steel mills. In this context, this study aimed to design, construct and validate a laboratory-scale system to investigate the yields of the three main coking products: coke, coal tar and coke oven gas (COG). Laboratory yields of coke, coal tar and COG, as well as the COG composition, were validated against industrial values obtained with the same coking blend. The apparatus, comprising a coking retort and a cracking column, was designed based on systems described in the literature. The coking process was carried out at 1000 °C with a heating rate of 3 °C/min. The optimal cracking temperature and residence time for reproducing industrial conditions were determined to be 800 °C and 30 min, respectively. Under these conditions, laboratory coke yields differed by 1.6% from industrial yields, while coal tar yields were equivalent. Average volumetric yields of COG were 341 Nm^3^/t, compared to 315 Nm^3^/t industrially. Methane and hydrogen concentrations, the two major gases in the COG, differed by less than 5%. The developed methodology demonstrates high repeatability and mass balance consistency (product recovery) ranging from 99.7 to 100.2%. These results confirm the system’s capability to replicate industrial conditions and provide a foundation for future studies on the impact of coals and additives on the yields and quality of coking products.

## Graphical abstract

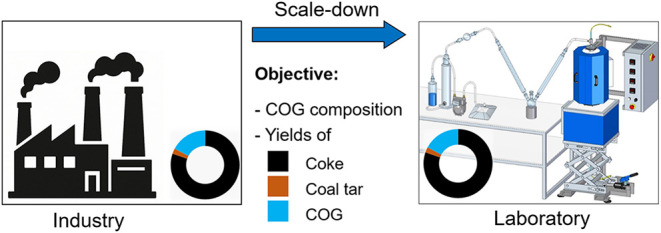

## 1 Introduction

Metallurgical coke, used as a reductant in blast furnaces, is a macroporous carbonaceous solid produced from the carbonization of specific rank coking coals at temperatures of about 1000 °C in the absence of oxygen.^1^ World coke production was estimated at 719.2 Mt in 2022, with Brazil being the seventh largest producer, contributing 1.3% (9.4 Mt) to the global production.^2^

There are two main coking processes: byproduct recovery and nonrecovery. Nonrecovery coke plants can utilize the gases to generate electric power and heat (heat-recovery). Byproduct coke plants, the subject of this study, are the most widely used, contributing over 90% of global coke production in 2017.^3^ The primary byproducts of the process are coke oven gas (COG) and coal tar.

COG is produced with a mass yield of about 15 to 20%.^4^ Due to its significant energy content, with a lower heating value in the range of 16 to 20 MJ/Nm^3^, COG has great energy relevance in the plant. Coal tar is produced with typical yields ranging from 3 to 5%.^3^ The recovered coal tar is separated by distillation into six fractions: five oils and a bottom stream, called coal tar pitch. These fractions are commercialized and represent an important revenue source for steel plants.

Data from the International Energy Agency indicates that steel production is responsible for 7% of anthropogenic CO_2_ emissions.^5^ Consequently, the steel industry has been under increasing pressure to reduce its emissions. In this context, the use of biomass or waste in coking represents a short-term alternative for replacing fossil fuels in steelmaking. However, this substitution requires an integrated scientific approach^6^ to allow a more comprehensive assessment of the impacts on technical and economic aspects across all process streams.

Koveria et al.^7^ reported that adding 5 wt % cellulose to four coking coals reduced their coking ability and swelling, mainly due to the release of oxygenated compounds, with dilatometry being the most sensitive test to detect these effects. Similarly, Bazaluk et al.^6^ found that 5% wood pellets increased CRI (34.0 to 36.9%) and decreased CSR (53.0 to 43.0%) of a coking blend. Fraga et al.^8^ showed that adding coal tar can compensate for the fluidity loss caused by biomass addition. The future implementation of such additions at the industrial scale should also consider their effects on coking byproducts.

The Iron and Steelmaking Laboratory (LaSid – *Laboratório de Siderurgia*) at the Federal University of Rio Grande do Sul was established in 1977, focusing on the SL/RN (Stelco–Lurgi/Republic Steel–National Lead) direct reduction process, which uses a rotary kiln to reduce iron ore with coal, producing sponge iron (DRI). Over the years, the group has worked on various topics related to steel industry, including studies on the thermoplastic properties of metallurgical coals and the quality of coke for blast furnaces.^9^ This study is part of a project aimed at expanding the laboratory’s research lines. The objective is to study the distribution and quality of coking byproducts from metallurgical coals and additives using the first laboratory-scale coking apparatus of its kind developed in Latin America.

The project consists of three phases: (i) the first phase involved the design and construction of a laboratory-scale experimental apparatus and the development of a methodology to reproduce industrial coking conditions, (ii) the second phase will investigate the individual behavior of coking coals and (iii) the third phase will evaluate the influence of additives typically studied for use in coking on the byproducts. This paper presents the results obtained in the first phase of the project, which aimed primarily to design and construct a coking apparatus based on a literature review.

A few laboratory-scale systems have been developed for coal coking studies, including the GOST 18635–73 apparatus, the Jenkner retort, and the KARBOtest. The GOST 18635–73 is a standardized setup, but its very small-scale limits flexibility, particularly for evaluating the effect of additives. The Jenkner retort is a classical reference and served as a basis for the design of the present apparatus. However, the versions described in the literature are mostly focused on coke evaluation, while detailed and consistent methodologies for assessing byproducts are not widely available. The KARBOtest is a modern commercial system, and although more advanced versions allow the evaluation of byproducts, detailed descriptions of the methods for their quantification and characterization are not available in the literature. In contrast, the system developed in this study allows the simultaneous evaluation of coke, coal tar, and COG, with a comprehensive description of its operating parameters that makes it adaptable to other operational settings in coking.

Based on this context, the present work first provides a brief conceptualization of the industrial coking process. Following an extensive literature review, including a discussion of the laboratory systems previously reported, the apparatus design and the development of the methodology are comprehensively described, grounded in the concepts presented earlier. Finally, validation experiments are presented, comparing the laboratory results with those obtained industrially for the same coking blend.

## 2 Industrial Coking Process and Recovery of Byproducts

### 2.1 Industrial Coking Process

A coking battery is a system composed of several vertical ovens interspersed with heating walls and combustion chambers. This design was introduced in the mid-1800s and has been improved over the years.^10^ The coal blend is loaded from the top through about 3 to 5 holes in the roof of the ovens,^11^ with a particle size distribution of about 80% below 3 mm and a moisture content of 7 to 10%.^3^ Heating is carried out by heating channels positioned between the coking chambers, operating at temperatures ranging from 1150 to 1350 °C.

A typical size for a coking chamber is 45 to 60 cm in width, 4 to 8 m in height, and 12 to 18 m in length.^12^ After charging, the charge quickly reaches the water evaporation temperature, causing the water to evaporate, migrate into the charge and condense on cold particles. As the process occurs continuously, this heat transfer mechanism keeps the charge at 100 °C until the coking front arrives.^13^

When the blend reaches a temperature of 350 °C, the plastic stage begins, where the coals soften and swell. As a result, there is a significant generation of gases and aromatic hydrocarbons that make up the primary tar. The volatiles are released primarily (about 70–90%) through the cracks in the layers of semicoke and coke and then rise through the space between the coke and the hot wall.^3^ Only a small portion of the volatiles migrate to the center of the oven, where they impregnate the coal particles in a manner similar to the water evaporation process described earlier.

As the vapors pass along the hot wall and through the empty space above the coal charge, the primary tar undergoes cracking reactions, or secondary reactions, resulting in a decrease in its yield accompanied by changes in its composition and properties. Cracking reactions, such as dealkylation, dehydrogenation and dehydroxylation also cause an increase in aromaticity, the destruction of aliphatic hydrocarbons, a decrease in phenol concentration and a reduction in the proportion of substituted aromatics.^14^ The secondary tar produced is also referred to as high-temperature coal tar.^15^

The plastic stage ends with the resolidification of the plastic mass at around 500–600 °C, resulting in the formation of semicoke. In the postplastic stage, above 600 °C, the growth of graphite layers in the semicoke occurs, which is accompanied by the release of hydrogen.^3^ The coking process typically lasts between 12 to 20 h.^16^ After the cycle is completed, the coke is removed from the oven with the aid of a coke pushing machine. The incandescent coke is then sent for cooling, also referred to as quenching or extinction.

### 2.2 Recovery of Coking Byproducts

In a byproduct coking plant, the vapors generated during coking are cooled by direct contact with a solution containing ammonia^17^ and then sent to the byproducts plant to obtain the following products: coke oven gas, coal tar and recovered ammoniacal liquor. Since the coke ovens are operated continuously and the cycles of each oven are started at different times, the raw streams are supplied to the byproducts plant without significant variations in composition.^18^

Figure 1 presents a typical flowchart of the coal tar and COG recovery process in byproducts coking plants. The raw COG exits the coking chamber through a riser pipe and enters the gooseneck, which connects to the collecting main by a valve.^19^ The same collecting main serves the entire battery, running over all the ovens parallel to the battery. In this collector, the hot raw gas (∼850 °C) is cooled by spraying ammoniacal liquor until it reaches temperatures below 85 °C, sufficient to be sent to the primary cooler.

**Figure 1:**
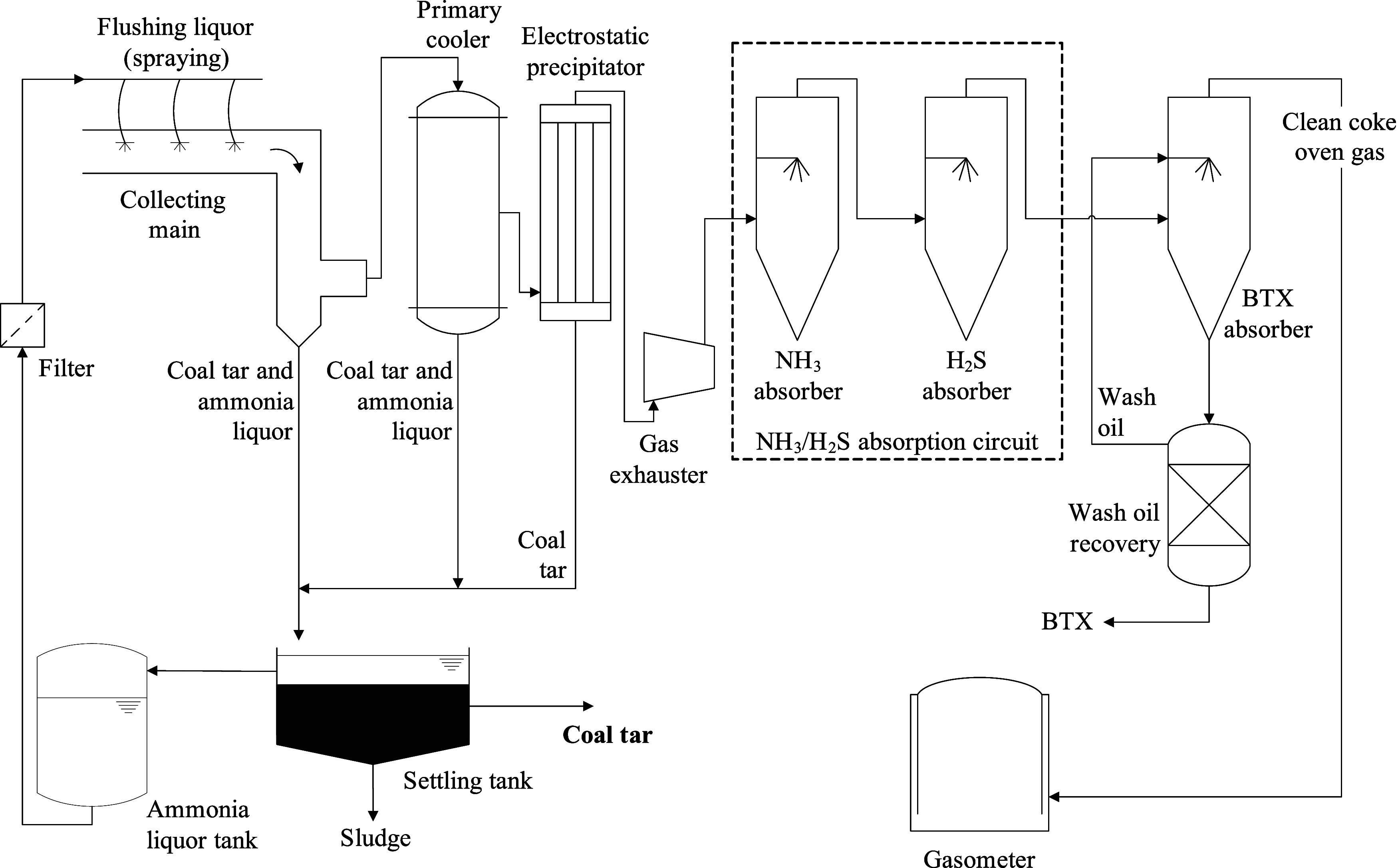
Process flowchart of the coal tar recovery and coke oven gas cleaning system in byproducts coking plants.

In the primary cooler, the gas is then cooled to temperatures below 35 °C, allowing the condensation of more coal tar and most of the water contained in the gas.^20^ Upstream of the exhaust fan, an electrostatic precipitator is used to remove tar aerosols from the gas.^21^ As illustrated in Figure 1, the condensate, composed of ammoniacal liquor and coal tar, is sent for separation in settling tanks. The ammoniacal liquor is recovered as the upper phase, while the denser tar is recovered and sent to the distillation plant. The decanter also serves for the sedimentation of fine solid materials carried with the coal tar.

After tar removal, the COG is sent to a circuit for the removal of ammonia and hydrogen sulfide, composed of well-established commercial processes.^21^ Ammonia (NH_3_) is an alkaline gas associated with corrosion problems in the recovery plant. Three main processes are used for its removal: (i) ammonium sulfate process, (ii) Phosam process and (iii) water scrubbing process. Hydrogen sulfide (H_2_S) is an acid gas that, in addition to causing corrosion and deposit formation in pipelines and equipment,^22^ also contributes to the formation of SO_*x*_ during the combustion of coke oven gas. The three types of commercial processes used for H_2_S removal are (i) dry oxidation processes, (ii) wet oxidation processes and (iii) absorption/scrubbing processes.

## 3 Laboratory Apparatus for Studying the Yields of Coking Byproducts

The study of coking byproducts requires a laborious and lengthy analysis involving the use of various equipment and reagents.^23^ The three different methodologies found in the literature for obtaining these yields are presented below. This review is essential for understanding the parameters and equipment used in each system, as well as their limitations, which are also briefly described. These concepts are fundamental to support the construction of the intended coking system.

### 3.1 GOST 18635–73 Standard (ΓOCT 18635–73)

The GOST 18635–73 standard “Hard coals: Method for determination of the yield of chemical coking products” is widely used in Russian and Ukrainian studies.^24^ The standard was introduced in 1974 by the Ministry of Ferrous Metallurgy of the Soviet Union and is currently maintained by the Euro-Asian Council for Standardization, Metrology, and Certification. The scheme of the system established by the standard for coking and quantification of products is presented in Figure 2.

**Figure 2:**
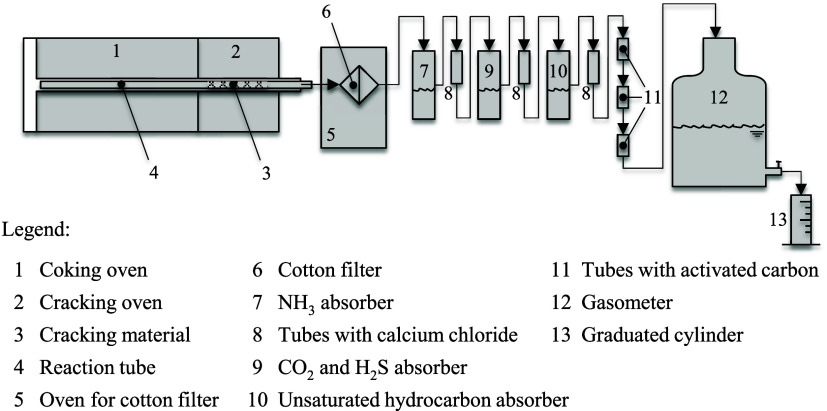
Apparatus for determining the yields of coking products according to the GOST 18635–73 standard.

According to the standard, the coking and cracking furnaces are preheated to temperatures of 240 ± 10 °C and 790 ± 10 °C, respectively. For the test, a 20 g sample of coal (<0.2 mm) is inserted into the coking oven, which is heated at a rate of 5 °C/min up to a final temperature of 890 ± 10 °C and maintained at this final temperature for 30 min. Yields are obtained by measuring the mass gain in the respective absorber solutions or through titration methods.

The volume of gas generated is obtained from the amount of solution flowing out of the gasometer during the experiment. The standard also recommends the use of another method, GOST 5439–76, for determining the gas composition. This method relies on selective absorption and selective combustion for quantifying the species.

### 3.2 Jenkner Retort

The Jenkner retort was introduced by Jenkner et al.^25^ to study the coking ability of coals, associated with the evaluation of the yields of coking byproducts. The configuration of the apparatus introduced by this study is shown in Figure 3. The retort has a capacity of 1.5 kg and its dimensions are not described. There is no cracking furnace for the generated vapors, which are immediately directed to a condenser. However, the coking furnace is preheated to 1000 °C before the insertion of the retort, creating a high-temperature region above the charge, which contributes to the cracking of the vapors.

**Figure 3:**
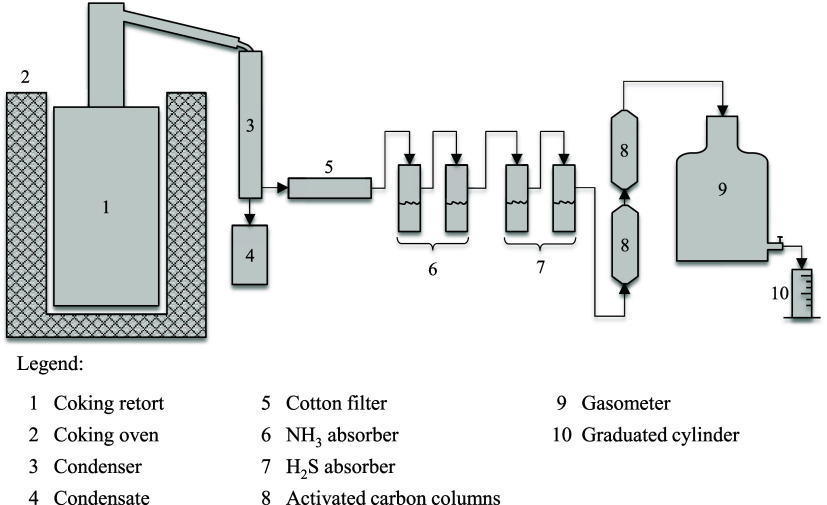
First arrangement of the Jenkner retort used to determine the yields of coking products.^25^

The condenser (3) is used to remove water and most of the coal tar. A cotton filter (5) is used to remove the remaining coal tar fraction. Ammonia is absorbed in two flasks containing a sulfuric acid solution (6), while the hydrogen sulfide is absorbed in two flasks containing a cadmium acetate solution (7). The coal tar retained in the filter is removed by pressing and the remaining portion is quantified by mass difference. The coal tar/water emulsion is separated in a separation funnel, followed by distillation.

The gas is collected in a gasometer (9), previously containing water. The volume of gas produced is measured by the volume of water that flows out (10) while maintaining a specific pressure range in the gasometer. The amount of gas generated in an experiment is sufficient to measure its heating value using a Junkers-type calorimeter.

Over the years, the Jenkner apparatus has been used, with minor modifications, by various research groups to study the yield of coking products.^26^ This system is also used by research groups in the steel industry, such as Tata Steel.^27^

Among the improvements introduced to the apparatus developed in 1934 are the addition of a cracking column above the coking retort and the use of an electrostatic precipitator. The cracking column is used to intensify the cracking of primary tar and simulate the process that occurs in industrial chambers, with the passage of vapors along the walls and through the upper zone of the chamber, both at high temperatures. The electrostatic precipitator is an improvement introduced for the removal of tar aerosols, although a small cotton filter is still used downstream of the precipitator.^26b,27^

One example is the apparatus presented by Loison et al.,^13^ which consists of a cylindrical retort and a cracking column filled with alumina beads, as shown in Figure 4. The authors suggest that the column’s operating temperature and the quantity of beads should be determined experimentally in order to reproduce the yields obtained in the industry. The system also includes an electrostatic precipitator and a series of glassware. Details of the sampling train can be found in Loison et al.^13^

**Figure 4:**
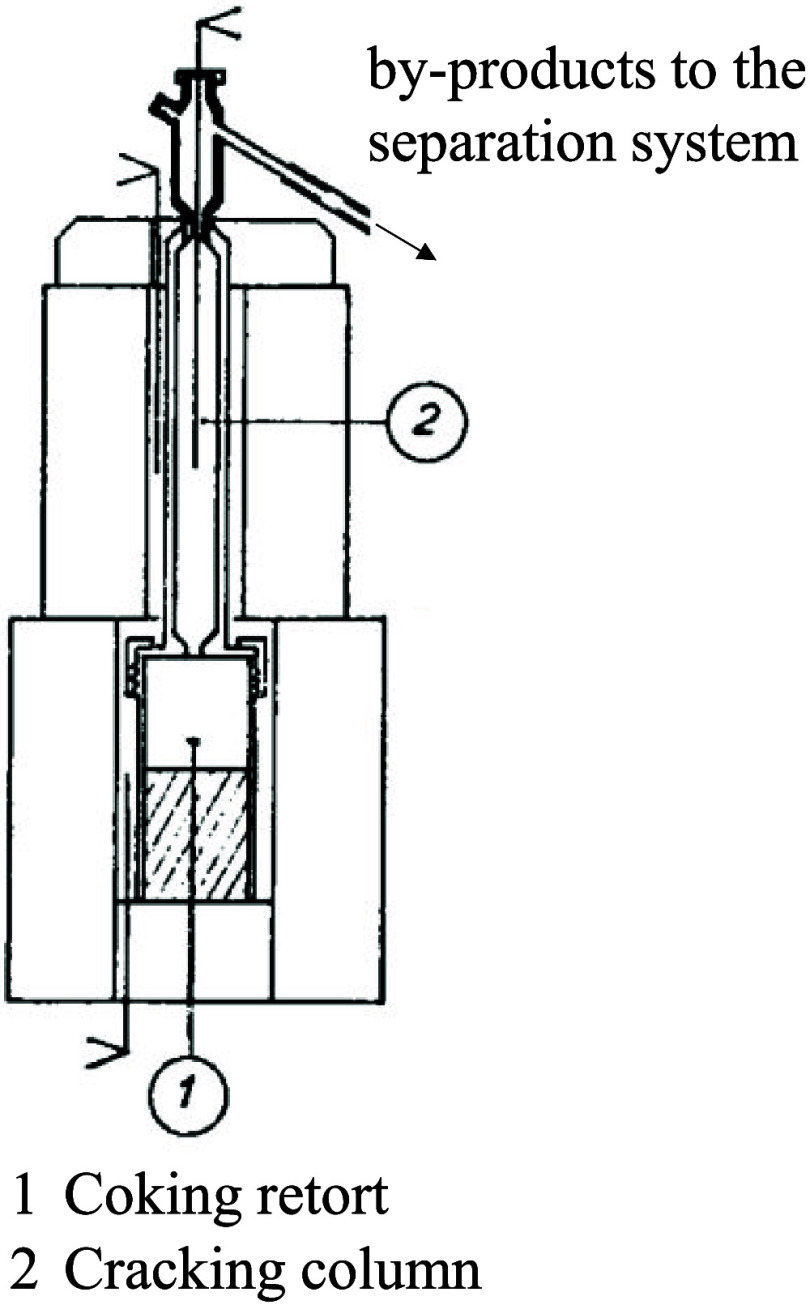
Schematic drawing of the Jenkner apparatus composed of coking retort and cracking column. Scale: 1:15.^13^ Reproduced with permission from Loison et al.^13^ Copyright 1989 Elsevier.

Although the dimensions of the assembly were not described by Loison et al.,^13^ the scale presented in Figure 4 allows for an estimation. Table 1 presents these dimensions, along with the dimensions of the retort from other Jenkner-type systems found in the literature. In addition to the retort dimensions, for reference, Table 1 describes the mass of coal used in the experiments and the charge density.

**Table 1:** Dimensions of Jenkner Retorts Described in the Literature

reference	height (mm)	diameter (mm)	charge mass (kg)	charge density (kg/m^3^)
Krebs et al.^26a^	260	120	1.0	740
Sutcu et al.^26b^	260	120	1.2	
Tiwari et al.^27^			1.2	750
Nomura and Nakagawa^28^	180	width: 35	0.1	
		length: 175		
Loison et al.^13^	139^a^	120	1.25	750

a Height of coal charge.

### 3.3 KARBOtest Apparatus

The KARBOtest apparatus is a commercial system developed by the Institute for Chemical Processing of Coal (IChPW – Instytut Chemicznej Przeróbki Węgla) to determine the quality of coals and coal blends for coke production, as well as to determine process yields. The system is used by various steel mills such as Třinec Iron and Steel Works (Czech Republic),^29^ ArcelorMittal Kryviy Rih (Ukraine), EVRAZ Nizhny Tagil (Russia),^30^ Cherepovets Steel Mill (Russia)^31^ and Altai-Koks coke plant (Russia).^32^

The system includes a retort with a diameter of 150 mm and a height of 850 mm, with a capacity for coking 4 kg of sample.^33^ This quantity is sufficient to obtain a mass of coke that allows reliable evaluation of its properties for quality control. For the coking experiment, the retort is inserted into the furnace and heated until the center reaches a temperature of 950 °C. The retort is maintained at this temperature until the gas flow produced is less than 0.5 L/min.^34^ Due to the larger dimensions of the retort, a cracking column is not used.

Table 2 presents a comparison among the described methodologies. The main advantage of systems operating with larger mass is the ability to assess the quality of the produced coke. This feature is particularly valuable in studies involving blends of metallurgical coals and additives such as biomass and residues. The Jenkner retort is partially limited in this advantage due to the quantity of coke produced (∼600 – 750 g). The smaller mass used in the GOST 18635 standard does not reflect a less complex system for operation, as detailed in Item 3.1.

**Table 2:** Comparison of Operation Parameters and Characteristics of Different Apparatus for Determining Coke Oven Byproducts

	method		
parameter	GOST 18635	Jenkner retort^25^	KARBOtest^35^
charge mass	20 g	∼1.5 kg	∼4.0 kg
coking time	2h40	2h40	Q_COG_ < 0.5 L/min
yields (coke, tar, and COG)	yes	yes	yes
yields (NH_3_, H_2_S, BTX)	yes	yes	yes^b^
coke quality evaluation	no	yes^a^	yes

a The amount of coke produced allows for a partial evaluation of coke quality.

b The more complete version allows for the assessment of the yield of these products.

## 4 Design of A Laboratory System for the Study of Coking Byproducts

Considering the characteristics of the equipment presented and discussed earlier, a system based on the Jenkner retort was constructed to meet the objectives of the present study. Moreover, the detailed project presented can serve as a basis for the development of additional equipment in research centers to support the growing demand for studies on renewable raw materials as alternatives to coal for metallurgical coke production. The works presented in the Table 1 were used, among others, as a reference for the design of the experimental apparatus. Based on these data, the dimensions of the retort were defined as 120 mm in diameter and 260 mm in height.

In this equipment, a cracking column is necessary to simulate the secondary cracking that occurs in industrial chambers, thus approximating the composition of the obtained tar. In the absence of detailed information about the cracking column, its design was carried out considering an L/D ratio of 10, with a useful length of 440 mm and a diameter of 44 mm. The scale of a diagram presented by Loison et al^13^ of a Jenkner retort allows for the estimation of cracking column dimensions similar to those established in this project. Considering that not the entire length of the column remains in the hot region of the furnace, since both the upper and lower parts of the column are in contact with the furnace insulation (cold region), the column was constructed with a total length of 560 mm.

The reactor was constructed from AISI 310 stainless steel due to its suitability for high-temperature applications, offering good resistance to oxidation and corrosion, as well as low scaling under repeated thermal cycles. The heating of the assembly is achieved using two independent furnaces. Thus, the design included a split furnace for heating the cracking column and a furnace with a lift for the coking retort, allowing for preheating with the retort outside the furnace (lowered furnace). Figure 5 shows the schematic drawing of the designed reactor. The furnace elevator is foot-operated, enabling safe movement even when the furnace is hot. Both furnaces were built with a power of 2.8 kW.

**Figure 5:**
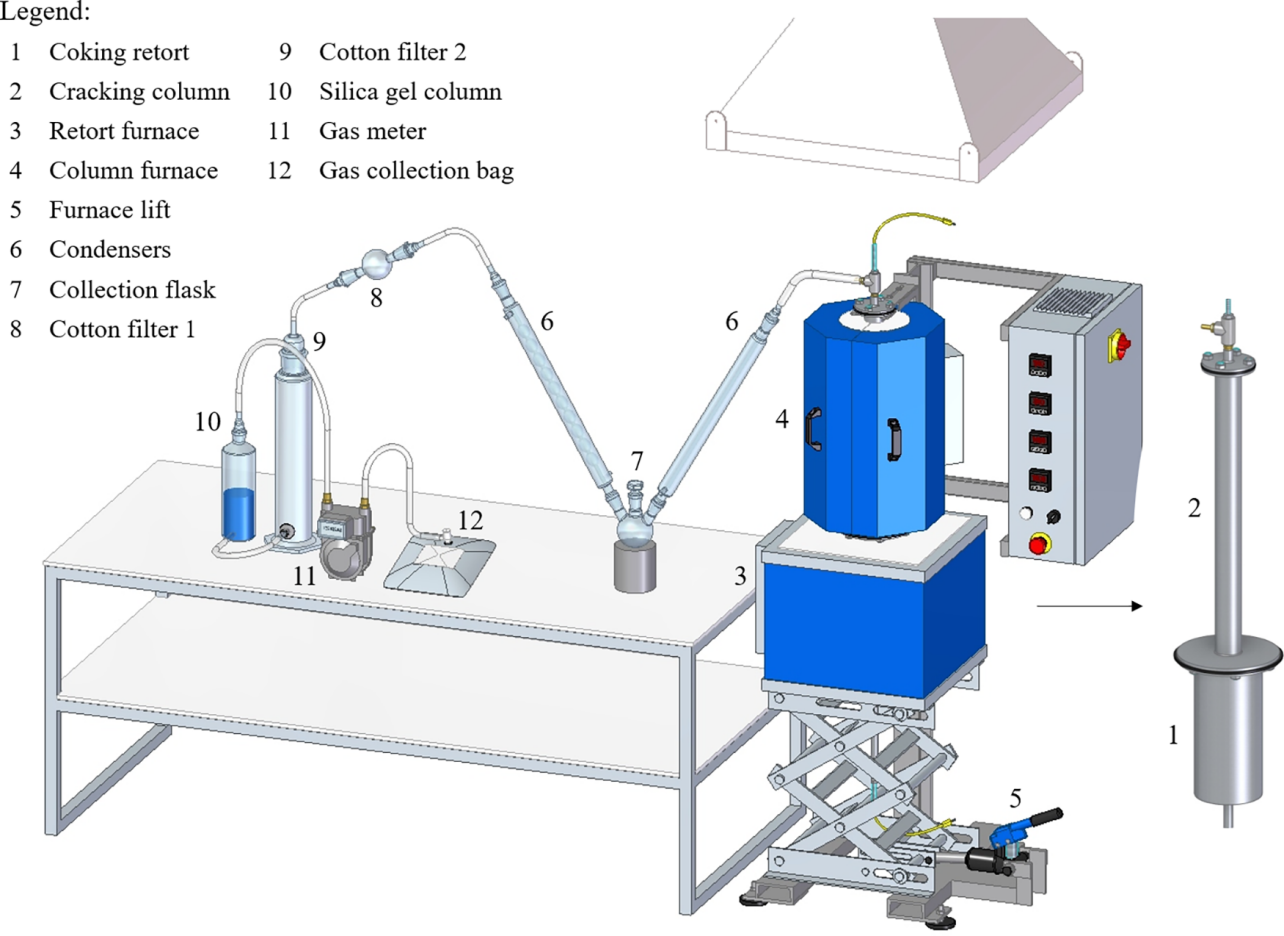
Drawing of the laboratory apparatus designed for coking experiments with byproduct recovery.

The system used for the collection of byproducts was also designed based on the studied sources and its schematic drawing is presented in Figure 6. Initially, it consists of two water-cooled condensers and a 250 mL flask for collecting the condensed tar. The cooling water was supplied by an ultrathermostatic bath (7Lab, model Bio Termo 20) maintained between 3 and 5 °C. The condensers act as the primary cooler used in the industry. As illustrated in the schematic, the first condenser used was a Liebig type, and an Allihn condenser was used at the outlet of the flask. The Allihn condenser was only used at the outlet of the collection flask because, despite its higher efficiency and lower risk of obstruction,^36^ it is more difficult to clean.

**Figure 6:**
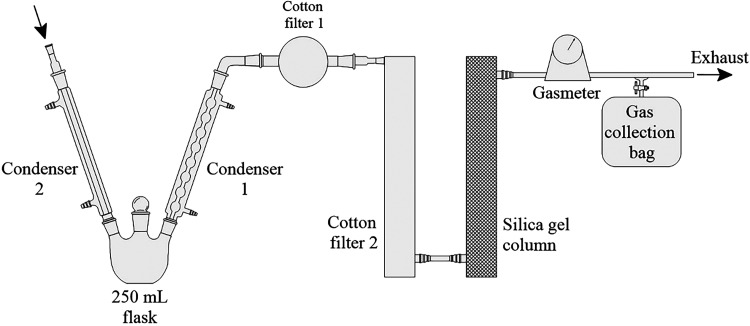
Schematic of the system for collecting tar and coke oven gas.

Given the complexity of operating and scaling down electrostatic precipitators used in the industry for aerosol abatement, cotton filters were used for the laboratory apparatus. Thus, two cotton filters were positioned after the second condenser. The first is a spherical filter with a diameter of 70 mm and the second is a cylindrical filter with a diameter of 90 mm and a height of 450 mm. A total of 3 and 60 g of cotton were used in filters 1 and 2, respectively. The cotton was previously dried at 105 °C for 2 h.

After the second filter, a column containing 400 g of silica gel beads, with diameters between 4 and 8 mm, was used to remove moisture from the COG. The dry, tar-free COG was sent to a diaphragm-type gas meter (LAO, model G1). Downstream of the meter, gas samples were collected in appropriate bags (SKC, model FlexFoil) at pre-established time intervals. The collection times were defined in pretests. Since there is minimal pressure drop in the laboratory collection train, there is no need for equipment to promote gas flow.

### 4.1 Coking Methodology

The equipment was designed for a feed of 1.0 kg of coking blend, resulting in a charge with a height of 110 mm and a density of 800 kg/m^3^. Considering the capacity of the coking plant at Gerdau Ouro Branco, the laboratory apparatus represents a scale-down of approximately 7 million times. The cracking column was filled with 500 g of alumina beads, resulting in a column height of 10 in (254 mm). Approximately 400 beads with a diameter between 8 and 10 mm were used, which were precalcined at 850 °C for 2 h. These beads were composed of α-Al_2_O_3_ with low surface area (<1 m^2^/g), chosen to favor the thermal cracking of vapors.

Initially, the retort furnace was heated to 550 °C and the cracking column was heated to 780 or 800 °C. At this stage, the retort was positioned outside the furnace while heating, whereas the cracking column with the alumina beads was inside its respective furnace. After reaching the established temperatures, the retort was quickly inserted into the furnace, resulting in a temperature drop due to the insertion of the cold retort. Once the temperature recovered to 550 °C, the furnace was heated to 1060 °C at a heating rate of 3 °C/min. This temperature was determined in pretests to ensure that the center of the charge reached 1000 °C.

Figure S1 shows the thermal profile of the coking furnace obtained by temperature measurements taken every 20 mm along the central axis of the assembly. Under the evaluated condition, 400 mm of the cracking column, which has a total length of 560 mm, was maintained at temperatures above 800 °C. Therefore, the column presents a large proportion of useful length to be employed for cracking. It should be noted that the thermal profile is modified when the system is operated during an experiment, with COG flow and alumina beads inside. Nevertheless, the measurement carried out with the empty setup provides useful information about the temperature distribution and the identification of hot and cold zones within the system. It was also observed that the temperature at the flange connecting the cracking column and the coking retort is around 790 °C, which is sufficiently high to avoid condensation of vapors generated in the retort.

In the first three experiments, two residence times were evaluated, 10 and 30 min, along with two cracking temperatures, 780 and 800 °C. The validation experiments were labeled as VN, where N represents the experiment number according to Table 4. Once the optimal parameters were defined to replicate industrial yields, triplicate experiments were conducted under these conditions to evaluate the repeatability of methodology and the composition of COG (V4, V5 and V6).

### 4.2 Methodology for Quantifying Coking Products

#### 4.2.1 Determination of Coke Yield

After the experiment concluded, the furnaces were turned off and the retort was cooled inside the furnace until it reached 500 °C. Then, the furnace was lowered and the retort was cooled in ambient air overnight. After discharge, the coke yield (*Y*_coke_) was obtained according to eq 1.

1 $$Y_{\mathrm{coke}}=\frac{\left(m_{\mathrm{coke},r}+m_{\mathrm{coke},c}\right)}{m_{\mathrm{blend}}}\times 100$$

where:

*m*_coke,r_ is the mass of primary coke, obtained in the coking retort (g),

*m*_coke,c_ is the mass of secondary coke, deposited on the alumina beads (g),

*m*_blend_ is the mass of coking blend on a dry basis (g).

#### 4.2.2 Determination of Coal Tar Yield

Coal tar quantification was carried out by adding two parts, following the strategy described in Figure 7. Coal tar recovered from glassware, tubing and cotton filters was obtained by mass difference. For the quantification of the other part of the tar, collected in the flask, a methodology was developed due to the high amount of water condensed simultaneously with the tar. Separation of water and tar by decantation, as occurs in industrial tanks, is difficult to achieve in the laboratory due to the small quantities and the difficulty of handling the tar without losses. To address this issue, the tar was extracted from the suspension by two extraction cycles with 20 mL of dichloromethane to separate the water. After this, the glassware was washed with acetone and all the recovered tar was sent for separation using a rotary evaporator to remove the solvents.

**Figure 7:**
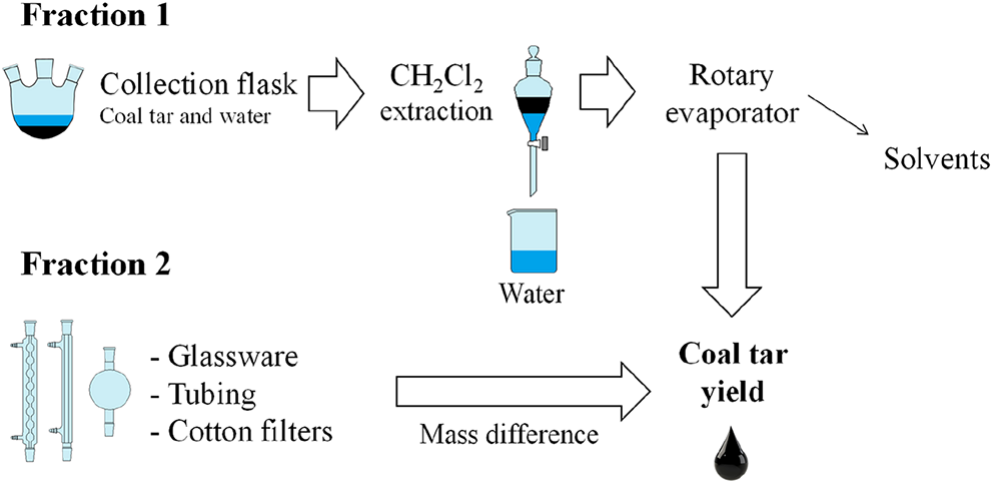
Schematic for quantifying coal tar produced in the coking experiments.

The evaporation of the solution containing dichloromethane, acetone and water, in addition to coal tar, was carried out using a rotary evaporator (Fisatom, model 803) at a temperature of 60 °C and absolute pressure of 35 kPa. After complete evaporation of the solvents, the remaining water was removed by adding 20 mL of anhydrous ethanol to the flask and evaporating at 70 °C and 20 kPa to carry away the residual water. This procedure was repeated until no more water was observed in the evaporation flask. Finally, the mass of the coal tar fraction recovered in the flask was obtained and the coal tar yield (*Y*_tar_) was calculated using eq 2.

2 $$Y_{\mathrm{tar}}=\frac{\left(m_{\mathrm{tar},1}+m_{\mathrm{tar},2}\right)}{m_{\mathrm{blend}}}\times 100$$

where:

*m*_tar,1_ is the mass of tar from fraction 1 (g),

*m*_tar,2_ is the mass of tar from fraction 2 (g),

*m*_blend_ is the mass of blend on a dry basis (g).

The water yield (*Y*_water_) was calculated from eq 3, by summing the water adsorbed in the silica gel and the water condensed in the collection flask. The mass of water in the flask was obtained by the difference between the total collected mass and the mass of coal tar obtained after evaporation. It noteworthy that the quantification of water by this methodology may include a small amount of light compounds evaporated under the conditions applied in the rotary evaporator.

3 $$Y_{\mathrm{water}}=\frac{\left(m_{\mathrm{water},1}+m_{\mathrm{water},2}-m_{\mathrm{water},\mathrm{blend}}\right)}{m_{\mathrm{blend}}}\times 100$$

where:

*m*_water,1_ is the mass of water condensed in the collection flask (g),

*m*_water,2_ is the mass of water obtained by the mass gain in the silica gel column (g),

*m*_water,blend_ is the mass of water contained in the coking blend (g),

*m*_blend_ is the mass of coking blend on a dry basis (g).

#### 4.2.3 Determination of the Composition and Properties of Coke Oven Gas

COG samples were analyzed using a gas chromatograph (Shimadzu, model GC-2014ATF/SPL) equipped with a thermal conductivity detector. Injections were performed through a 10-way valve with a 500 μL loop, maintained at 70 °C. Approximately 4 to 5 mL of samples were introduced into the valve using a gastight syringe (Hamilton, model #1005).

The chromatograph is equipped with a precolumn HayeSep Q 80/100 (1.8 m long, 2 mm id, Restak) and a separation column Carboxen-1000 60/80 (4.6 m long, 2.1 mm id, Supelco). Helium was used as the carrier gas at a flow rate of 30 mL/min. The injector and detector were operated at 200 and 240 °C, respectively. After the injections, the columns were maintained at 35 °C for 2 min, heated at 20 °C/min to 225 °C, and held at 225 °C for 12.5 min, totaling 24 min of analysis. Calibration curves were constructed using a primary standard gas (White Martins).

The concentration (*C_i_*) of each gas (*i*) in the COG was calculated according to eq 4, considering the concentrations obtained at each time interval by gas chromatography and the gas volumes in each of the eight time intervals (*j*) analyzed.

4 $$C_{i}=\frac{\sum_{j=0}^{8}\left[\left(V_{j}-V_{j-1}\right)\times \left(\frac{C_{i,j}+C_{i,j-1}}{2}\right)\right]}{V_{T}}$$

where:

*V*_*j*_ and *V*_*j‑*1_ are the cumulative gas volumes produced up to time *j* and *j-*1, respectively (Nm^3^),

*C*_*i,j*_ and *C*_*i,j‑*1_ are the concentrations of gas *i* at time *j* and *j-*1, respectively, obtained by gas chromatography (vol %),

*V*_*T*_ is the total volume of coke oven gas (Nm^3^).

From the composition, the lower heating value (LHV, in MJ/Nm^3^) and density (ρ, in kg/Nm^3^) of coke oven gas were calculated using eqs 5 and 6, respectively, based on the properties of the pure species shown in Table S1.^37^

5 $$\mathrm{LHV}=\frac{\sum \left(\frac{C_{i}}{100}\times {\mathrm{LHV}}_{i}\right)\times 0,004184}{0,0224}$$

6 $$\rho =\sum \left(\frac{C_{i}}{100}\times \rho_{i}\right)$$

where:

*C*_*i*_ is the concentration of gas *i* (vol %),

LHV_*i*_ is the lower heating value of gas *i* (kcal/mol), according to Table S1,

ρ_*i*_ is the density of gas *i* (kg/Nm^3^), according to Table S1.

After determining the yields of all products, the consistency of the mass balance of each experiment can be calculated by the ratio of the mass of the products to the mass of coking blend fed on a wet basis (*m*_blend,wb_), according to eq 7.

7 $$\mathrm{mass}\;\mathrm{balance}\;\mathrm{consistency}=\frac{m_{\mathrm{coke}}+m_{\mathrm{tar}}+m_{\mathrm{water}}+\left(V_{\mathrm{COG}}\times \rho_{\mathrm{COG}}\right)}{m_{\mathrm{blend},\mathrm{wb}}}\times 100$$

## 5 Validation of Laboratory Apparatus with Industrial Data

### 5.1 Validation Data

Validation experiments were conducted with a coking blend typically used in the industry. The blend was provided by Gerdau Ouro Branco and has the following composition: 50% medium-volatile bituminous coals, 20% high-volatile bituminous coals and 30% green petroleum coke. The batch has an ash yield percentage of approximately 7 wt % and a volatile matter content of 21 wt %. The components were used in validation experiments in the same granulometry as in the industry (80% < 3 mm) and with the moisture content held at 8 wt %.

The yields obtained from the industrial coking of this sample, presented in the Table 3, were provided by the company and used for the validation of the laboratory apparatus. Although the industrial yields fall within a typical range, variations are mainly observed due to the composition of the coking blend used by each plant, as well as differences in process parameters. The developed system can be calibrated for any case following the same methodology presented here.

**Table 3:** Industrial Reference Coking Yield and Comparison with Other Industrial Yields

product	Gerdau Ouro Branco (reference)	PAO Alchevskkoks^38^	PAO Koks^39^
coke (wt %)	80.3	77.2	76.1
coal tar (wt %)	2.5	4.7	4.1
COG (Nm^3^/t)	300–330		
COG (wt %)	12.3	18.1^a^	15.5
water (wt %)	4.9^a^		3.5

a Calculated by difference.

### 5.2 Determination of Laboratory Coking Parameters

Figure 8 shows the complete system during a coking experiment where coal tar retention is observed along the glassware, flask and filters. At the end of the experiment, it was found that a large portion of the cotton in Filter 2 is still clean, indicating that the tar-free coke oven gas exits the filter from the bottom.

**Figure 8:**
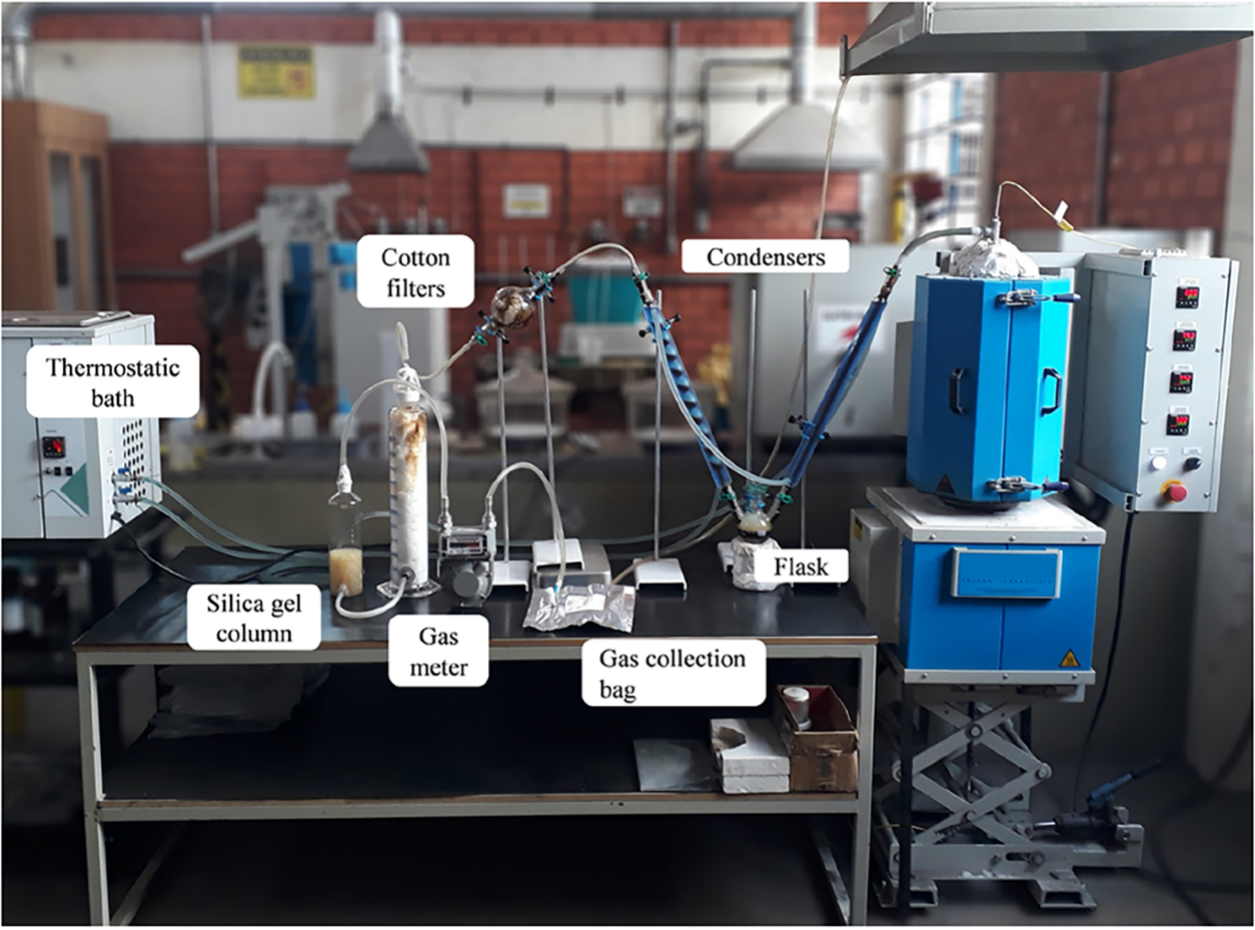
Experimental setup configured for the validation experiments.

Figure 9 shows the typical temperature profile for the coking experiments. Data were collected every 1 min for the first 10 min and every 10 min thereafter. After inserting the retort into the preheated furnace, a rapid temperature drop in the furnace to about 430 °C was observed, followed by a recovery to the initial temperature after approximately 8 min.

**Figure 9:**
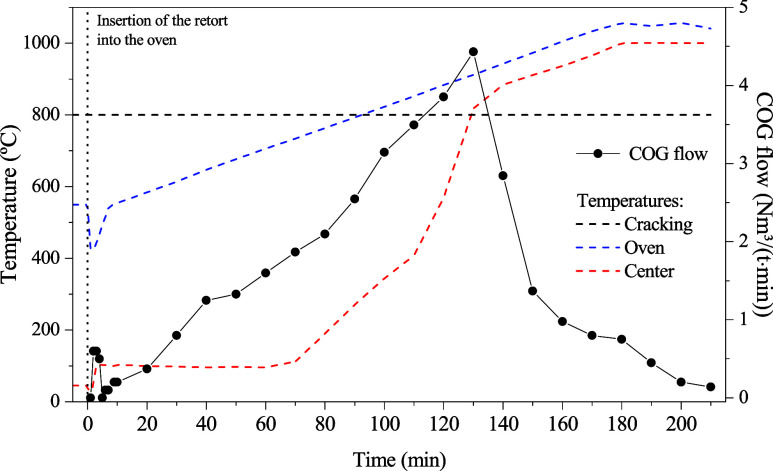
Temperature profile of the retort and cracking column during coking and volume of coke oven gas generated throughout experiment V2.

Similar to what happens in industry, the center of the charge remained close to 100 °C, the water evaporation temperature, until the coking front arrived. After the coking front reached the center of the retort (∼70 min), a high heating rate of up to 25 °C/min was observed until a temperature difference of 60 °C compared to the furnace (at 900 °C) was reached. This difference was maintained until the end of the experiment at 1000 °C, following the furnace heating rate of 3 °C/min.

The volume of clean COG produced was recorded at the same time intervals as the temperature. The flow rate was determined by the gas volume generated in each time interval, and the results are also shown in Figure 9. It can be observed that the peak of COG generation (4.4 NL/min) occurs when the retort center reaches approximately 820 °C.

The yields of the first three experiments are presented in Table 4. Coke yields similar to, but slightly higher than, industrial yields were obtained, with a difference of up to 1.5%. The higher yield obtained is associated with controlled laboratory conditions, where there is no combustion of the coke during discharge.^13^ For example, Stepanov and Karpin^40^ estimated coke losses of about 0.5%/min in the quenching car. Of the yield of approximately 82%, the coke removed from the retort and the cracking column contributes about 81% and 1%, respectively. Additionally, there was only a small difference in coke yield with the increase in soaking time from 10 to 30 min.

**Table 4:** Influence of the Studied Parameters on the Laboratory Yields of Coking Products

		experiments		
parameter/yield	reference (industrial)	V1	V2	V3
**parameters**				
coking blend mass (kg)		1.0	1.0	1.0
cracking temperature (°C)		800	800	780
soaking time (min)		10	30	30
**yields (dry basis)**				
coke (wt %)	80.3	81.8	81.7	81.5
coke oven gas (Nm^3^/t)	300–330	340.3	345.8	323.7
coal tar (wt %)	2.5	2.6	2.4	3.4
water (wt %)		0.7^a^	4.2	4.0

a Experiment conducted without the silica gel column.

The coal tar yield is also similar to the industrial yield for both soaking times evaluated. The residence time at 1000 °C of the coke does not influence the tar yield since there is no tar release during secondary devolatilization. On the other hand, an increase in the generation of COG was observed.

Despite the increase in COG generation and deviation from the reference yield, the longer residence time caused a reduction in the specific flow rate at the end of the experiment, from 0.38 to 0.13 Nm^3^/(min·t). This criterion is used in literature studies, by the KARBOtest apparatus, to define the end of coking in laboratory systems, when the specific flow rate reaches values below 0.13 Nm^3^/(min·t).^32,33^ Following this criterion, a soaking time of 30 min was defined.

Decreasing the cracking temperature to 780 °C (V3) increased the coal tar yield to 3.4%, which is 36% higher than the reference value. This increase in coal tar yield was accompanied by a decrease in COG yield from 349.3 to 323.7 Nm^3^/t. Consequently, it was observed that the yields of coal tar and COG are strongly dependent on the column temperature, which directly influences the severity of the cracking process.

Based on the evaluation, experimental parameters V2, featuring a 30 min soaking time and an 800 °C cracking temperature, were identified as optimal for reproducing the reference industrial condition. The higher gas yield (5.8%) compared to the reference yield may be related to two factors: (i) the contribution of ammonia and hydrogen sulfide, which are not separated in the current configuration and are accounted for with the COG, potentially contributing up to about 7 Nm^3^/t, and (ii) the controlled laboratory conditions that allow for minimal losses in the collection of COG.

### 5.3 Evaluation of the Repeatability of the Laboratory Apparatus

After defining the V2 parameters as optimal for reproducing industrial conditions, three additional experiments were conducted under the same parameters. The experiments were performed in triplicate to assess experiment repeatability and to characterize the COG according to the methodology described in Section 4.2.3. The results obtained for the yields were similar to those achieved in V2 and are presented in Table 5. Deviations of 0.2 wt % were observed for coke, tar, and water yields, and 4.9 Nm^3^/t for COG. Consistencies in the mass balance close to 100% indicate minimal losses in the experiments.

**Table 5:** Comparison of Average Laboratory Yields with Reference Industrial Yields

		experiments				
yields (dry basis)	reference (industrial)	V4	V5	V6	average	standard deviation
coke (wt %)	80.3	81.5	81.8	81.4	81.6	0.2
coke oven gas (Nm^3^/t)	300–330	335.8	340.6	345.5	340.7	4.9
coal tar (wt %)	2.5	2.6	2.4	2.4	2.5	0.1
water (wt %)		4.2	3.9	4.3	4.2	0.2
mass balance consistency (%)		99.7	100.0	100.2		

The coke produced in the repetitions showed a volatile matter content of 2.0 ± 0.1 wt %, very similar to industrial coke, which has a volatile matter content of 1.8 wt %. The low volatile matter content of the laboratory coke corroborates that the established coking time is adequate. The low deviations obtained demonstrate the consistency of the developed methodology and also contribute to the differentiation of yields obtained from different coking coals and additives.

Table 6 presents a comparison of the ultimate analysis of coke and coal tar. The coke exhibited an elemental composition that is very similar and typical for metallurgical coke. Greater differences were observed in the laboratory coal tar, which showed slightly lower carbon concentrations and higher hydrogen concentrations. As a result, lower C/H molar ratios were observed for the laboratory coal tar (1.30) compared to the industrial coal tar (1.62), indicating a tar with lower aromaticity under laboratory conditions (i.e., less heavy fractions). More detailed characterizations are necessary to further clarify the significance of these differences.

**Table 6:** Comparison of Ultimate Analysis of Coke and Coal Tar Produced in Laboratory and in Industry

	ultimate analysis (wt %)				
sample	C	H	N	S	O
**coke**					
industrial	97.6	0.2	1.1	0.6	0.5
V5	97.6	0.4	1.1	0.4	0.5
**coal tar**					
industrial	92.3	4.8	1.1	0.6	1.2
V4	89.5	5.8	1.2	1.0	2.5
V5	90.0	5.7	1.2	0.9	2.2
V6	89.8	5.8	1.2	0.9	2.4

Figure 10 shows the comparison between the COG from laboratory experiments and the composition of industrial COG. Similar values to those found in industry were obtained for the concentrations of hydrogen and methane, which are the two major gases (∼90 vol.%) in the composition of COG. Regarding the average values, a gas with hydrogen and methane concentrations of 68.1 and 23.6 vol.%, respectively, was obtained, compared to 64.6 and 22.7 vol.% for the gas produced at the plant. Consequently, very similar H_2_/CH_4_ molar ratios were achieved between laboratory COG (2.88) and industrial COG (2.84).

**Figure 10:**
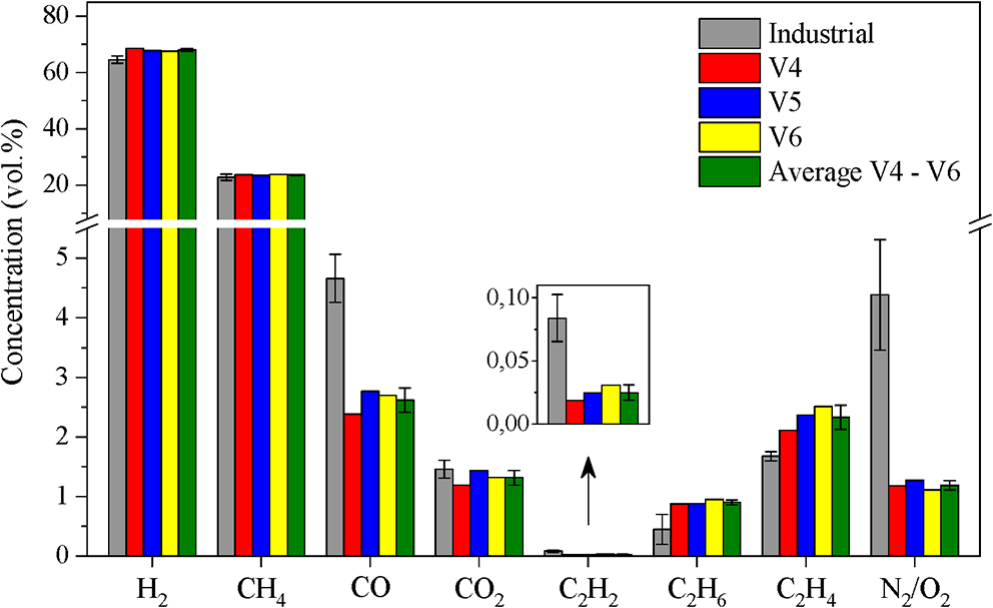
Comparison of the composition of coke oven gas obtained in the laboratory with the reference composition of industrial coke oven gas.

Regarding the other gases, a considerable difference was observed for carbon monoxide, with concentrations 46% lower in laboratory conditions. While the expected concentration is in the range of 5 to 8 vol.%,^21^ it is unclear why lower concentrations of carbon monoxide (2.6 ± 0.2 vol.%) were obtained in the laboratory. Additionally, lower concentrations of nitrogen and oxygen were observed in laboratory conditions. Due to the controlled laboratory conditions, it was possible to achieve less contamination of the COG with atmospheric air compared to the industrial setting, which shows concentrations in the range of 3 to 6 vol.%. Nonetheless, a small concentration (∼1 vol.%) was detected in all experiments.

Differences in air ingress between the systems may have caused the discrepancy in CO concentrations. In the laboratory retort, the apparatus is perfectly sealed, and the small amount of air contamination likely occurs only in the gas collection line (such as filters and condensers), where the COG is already cooled, thus preventing oxidation reactions. In contrast, in industrial operation, air may infiltrate into the coke oven furnace or other hot regions, where it can oxidize coke or methane present in industrial COG, resulting in slightly higher CO concentrations. This interpretation is supported by the N_2_:O_2_ ratio measured in industrial COG (95:5), which is considerably higher than that of ambient air (79:21), indicating that part of the oxygen entering the system is consumed by oxidation reactions.

The concentrations of C_2_H_*y*_ hydrocarbons followed the same trend observed in industrial COG: C_2_H_6_ > C_2_H_4_ > C_2_H_2_. Ethane and ethene showed concentrations slightly above those of industrial ones, while ethyne showed lower concentrations. The total concentrations of these compounds resulted in average C_2_H_*y*_ concentrations of 3.3 and 2.2 vol.% for laboratory and industrial conditions, respectively.

Figure 11 presents the main properties of coke oven gas. The gas produced in the laboratory has a lower density and slightly higher heating value compared to industrial gas. These variations are due to the minor differences in composition discussed earlier. The lower density is primarily due to the higher concentration of hydrogen (a light gas) and the lower concentration of air (heavy gases). The increased heating value is attributed to the higher concentration of hydrocarbons, particularly C_2_H_*y*_, as well as the reduced dilution of the COG with air.

**Figure 11:**
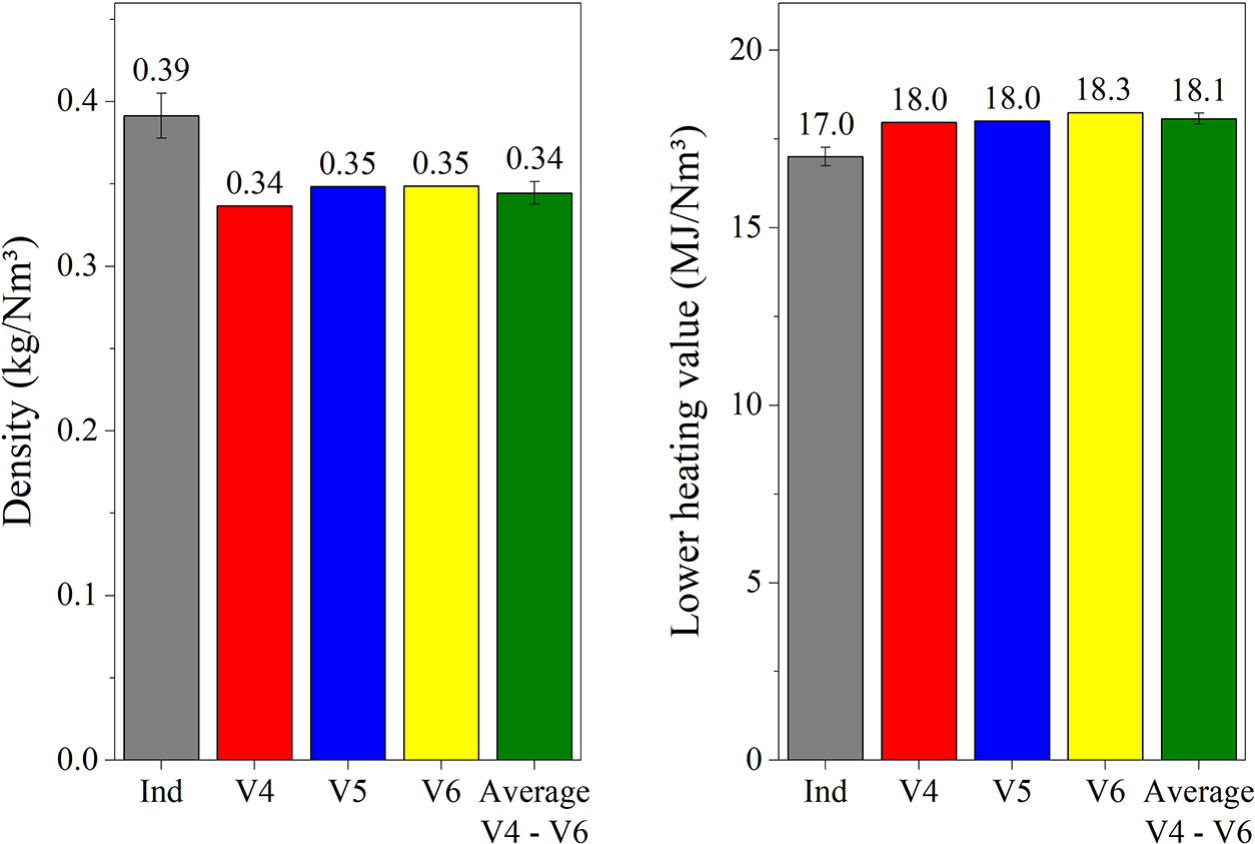
Comparison of the density and heating value of coke oven gas obtained in the laboratory with the reference industrial coke oven gas.

The coking process is highly complex, and the present experimental system is limited to reproducing the phenomena that occur specifically within the coking reactor. Other industrial effects, such as external air ingress, deposition of solids on the coke oven roof, elutriation of fines, or losses during coke quenching, cannot be reproduced at the laboratory scale and may influence the overall mass balance. These are examples of scale-dependent effects that are inherently absent in small-scale setups. Moreover, laboratory systems cannot fully replicate the thermal gradients and coking profiles observed in industrial coke ovens. Such effects must therefore be assessed under industrial conditions or in experimental setups specifically designed for this purpose.

Despite these limitations, the low deviations obtained, especially for the major gases, demonstrate high repeatability of the methodology in evaluating COG composition as well. Finally, sufficiently small differences were observed between laboratory and industrial results, thereby validating the system for studying the composition and properties of coke oven gas generated from various raw materials, such as hard and soft coals, and additives.

## 6 Conclusions

A brief conceptualization was presented, providing the basis for the reactor design. The design of the apparatus was described in detail and comprehensively. The results obtained in this study validated the laboratory-scale equipment for accurately replicating industrial coking conditions. The developed methodology proved suitable for determining the yields of tar and COG, as well as coke, due to its high repeatability. Additionally, the values obtained for the composition and properties of the COG were consistent and appropriate. Satisfactory results were obtained when comparing these values with the yields from industrial coking of the same coking blend. Differences in coke and coal tar yields were observed to be less than 2% compared to industrial yields. For COG, volumetric yields showed a difference of 3.2%.

Therefore, it is concluded that the developed laboratory-scale coking system is suitable for evaluating the contribution of individual coals and additives to the yield and quality of coking products. The results obtained with this system can directly benefit industrial coking by providing a better understanding of how different coals and additives influence the production of coal tar and coke oven gas. Such knowledge is particularly relevant for improving the value in use of coals in blending strategies and for assessing the impact of additives, especially under the increasing pressure to incorporate renewable, biomass-based materials in coking operations to reduce emissions. Future phases of this research will focus on the evaluation of coals typically used in Brazilian coking blends, including three high-volatile bituminous coals, three medium-volatile bituminous coals, and a Brazilian coking coal. Furthermore, the influence of three different additives, namely charcoal fines, green petroleum coke, and waste tires, will be examined in detail, with particular attention to their impact on coking byproducts.

## Supplementary Material



## Notes

Table with lower heating value and density of gases present in COG used for calculating COG properties; temperature profile of the retort/cracking column assembly (PDF)
