# Supplementary material for: Design and Validation of a Laboratory-Scale System for Investigating Coking Byproducts
Source: ACS Omega. 2025 Oct 16;10(42):50163–75. doi: 10.1021/acsomega.5c06722 (PMC12573177; doi:10.1021/acsomega.5c06722)
Supplement: Supplementary file 1 [file ao5c06722_si_001.pdf]

DESIGN AND VALIDATION OF A LABORATORY-SCALE SYSTEM FOR INVESTIGATING COKING BY-PRODUCTS

CHRISTIAN MANERA[a,c], GUILHERME LIZIERO RUGGIO DA SILVA[b], BRUNO DEVES FLORES[a], EDUARDO OSÓRIO[a], MARCELO GODINHO[c]*, ANTÔNIO CEZAR FARIA VILELA[a]

[a]Graduate Program in Mining, Metallurgical and Materials Engineering, Federal University of Rio Grande do Sul (UFRGS), Porto Alegre, Rio Grande do Sul, Brazil.

[b]Gerdau Ouro Branco, Ouro Branco, Minas Gerais, Brazil.

[c]Graduate Program in Process Engineering and Technologies, University of Caxias do Sul (UCS), Caxias do Sul, Rio Grande do Sul, Brazil.

*Corresponding author: Rua Francisco Getúlio Vargas, 1130, ZIP CODE: 95070-560, Caxias do Sul, RS, Brazil - Tel.: +55 54 3218 2100; E-mail: mgodinho@ucs.br

SUPPORTING INFORMATION

Table S1 – Lower heating value and density of the gases present in the coke oven gas [37]

Gas	PCI (kcal/mol)	ρ (kg/Nm^3)
H_2	57,80	0,089
CO	67,64	1,250
CH_4	191,76	0,716
CO_2	-	1,964
C_2H_2	341,26	1,162
C_2H_4	316,20	1,252
C_2H_6	300,10	1,342
N_2/O_2	-	1,292

Figure S1 – Temperature profile of the retort/cracking column assembly. Retort furnace: 1050 °C. Cracking column furnace: 850 °C

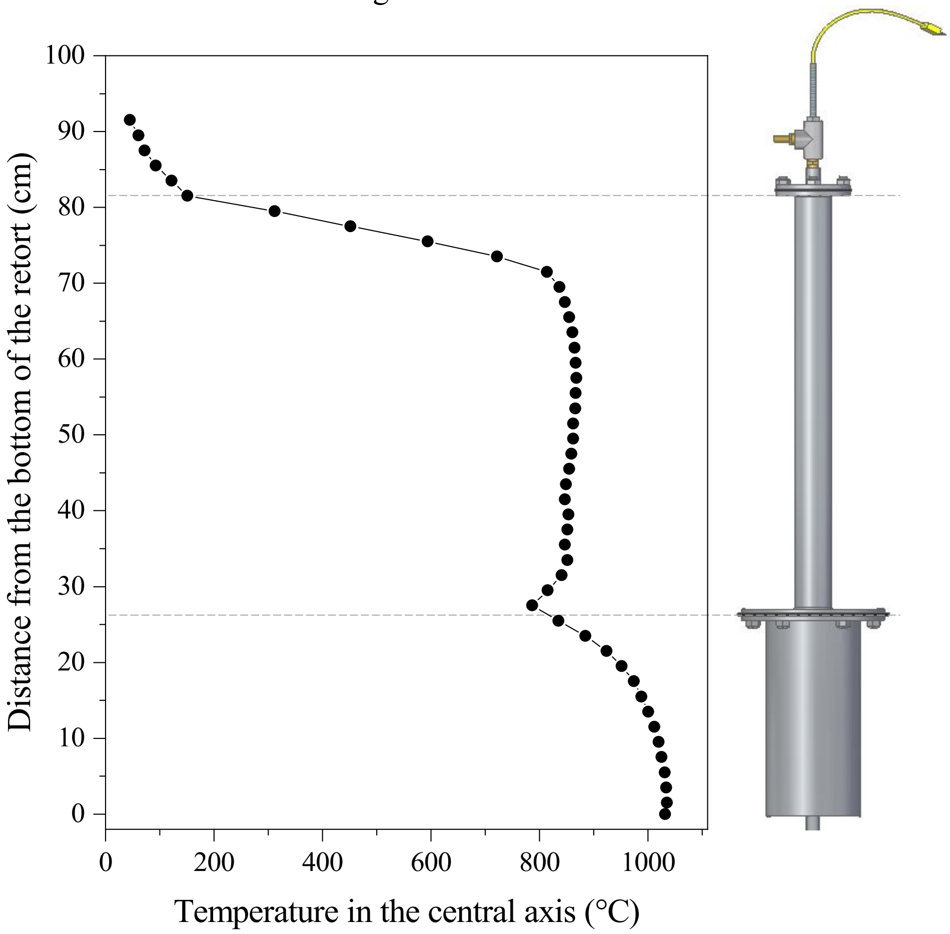

REFERENCES

(37) Perry, R. H.; Green, D. W., *Perry's Chemical Engineers' Handbook.* 8 ed.; McGraw-Hill: New York, 2008; p 2400 p.
